# Pharmacology of Herbal Sexual Enhancers: A Review of Psychiatric and Neurological Adverse Effects

**DOI:** 10.3390/ph13100309

**Published:** 2020-10-14

**Authors:** Pietro Brunetti, Alfredo Fabrizio Lo Faro, Anastasio Tini, Francesco Paolo Busardò, Jeremy Carlier

**Affiliations:** 1 Unit of Forensic Toxicology, Section of Legal Medicine, Department of Excellence of Biomedical Sciences and Public Health, Marche Polytechnic University, 60126 Ancona, Italy; pietrobrunetti40@gmail.com (P.B.); fabriziolofaro09@gmail.com (A.F.L.F.); jerem.carlier@gmail.com (J.C.); 2 Unit of Forensic Toxicology, Section of Legal Medicine, Department of Anatomical, Histological, Forensic, and Orthopedic Sciences, Sapienza University of Rome, 00198 Rome, Italy; anastasio.tini78@gmail.com

**Keywords:** aphrodisiac, sexual enhancer, plant, pharmacology, toxicology, psychiatry, neurology, adverse effect

## Abstract

Sexual enhancers increase sexual potency, sexual pleasure, or libido. Substances increasing libido alter the concentrations of specific neurotransmitters or sex hormones in the central nervous system. Interestingly, the same pathways are involved in the mechanisms underlying many psychiatric and neurological disorders, and adverse reactions associated with the use of aphrodisiacs are strongly expected. However, sexual enhancers of plant origin have gained popularity over recent years, as natural substances are often regarded as a safer alternative to modern medications and are easily acquired without prescription. We reviewed the psychiatric and neurological adverse effects associated with the consumption of herbal aphrodisiacs *Areca catechu* L., *Argemone Mexicana* L., *Citrus aurantium* L., *Eurycoma longifolia* Jack., *Lepidium meyenii* Walp., *Mitragyna speciosa* Korth., *Panax ginseng* C. A. Mey, *Panax quinquefolius* L., *Pausinystalia johimbe* (K. Schum.) Pierre ex Beille, *Piper methysticum* G. Forst., *Ptychopetalum olacoides* Benth., *Sceletium tortuosum* (L.) N. E. Brown, *Turnera diffusa* Willd. ex. Schult., *Voacanga africana* Stapf ex Scott-Elliot, and *Withania somnifera* (L.) Dunal. A literature search was conducted on the PubMed, Scopus, and Web of Science databases with the aim of identifying all the relevant articles published on the issue up to June 2020. Most of the selected sexual enhancers appeared to be safe at therapeutic doses, although mild to severe adverse effects may occur in cases of overdosing or self-medication with unstandardized products. Drug interactions are more concerning, considering that herbal aphrodisiacs are likely used together with other plant extracts and/or pharmaceuticals. However, few data are available on the side effects of several plants included in this review, and more clinical studies with controlled administrations should be conducted to address this issue.

## 1. Introduction

Sexual drive is influenced by biological, psychological, and social factors, but it can also be affected by medications and medical conditions. Many prescription drugs and narcotics (e.g., antidepressants, anxiolytics, antihistamines, antihypertensives, adrenergic receptor blockers, antipsychotics, and opioids) can negatively impact sexual desire, inhibit erection, ejaculation, or orgasm, and so on. Contrariwise, many aphrodisiac substances can improve sexual performance. In particular, substances of natural origin have been used worldwide for millennia in traditional medicines to boost sexual desire, sexual pleasure, or sexual behavior [1], and the use of psychoactive and/or stimulant drugs during intercourse, i.e., chemsex, is on the rise [2]. Nowadays, sexual performance anxiety, which contributes to psychogenic erectile dysfunction, is estimated to affect 9 to 25% of men in the United States, and phytotherapy is often employed as a treatment [3]. In 2007, approximately 56% of infertile couples had sought medical care worldwide [4] and many of these couples had opted for supportive complementary and alternative medicines to treat infertility. In 2010, in the United States, approximately 29% of 428 infertile couples had utilized an alternative treatment after 18 months of observation, 59% of which had taken herbal therapy [5]. Natural substances are mistakenly believed to be a safer alternative to modern medications with no side effects. They are also readily accessible on the Internet and specialized markets without a prescription. Consequently, the use of herbal supplements to enhance sexual drive has become increasingly popular, and more than 300,000 intoxications were reported to poison control centers over the last 20 years [6,7].

According to Sandroni, aphrodisiacs can be classified into three categories according to whether they increase potency (i.e., effectiveness of erection in males), sexual pleasure, or libido (i.e., sexual desire) [8]. Potency-enhancing substances typically induce vasodilation, often through the nitric oxide (NO) pathway, inducing hypotension and potentially harmful cardiovascular effects (e.g., sildenafil and L-arginine). Sexual pleasure-enhancing substances cause tumescence and lubrication of the genital mucosa, therefore increasing sensation (e.g., cantharidin). Libido-enhancing substances alter the concentrations of specific neurotransmitters (e.g., dopaminergic and serotoninergic pathways) or sex hormones (e.g., pituitary hormones and testosterone) in the central nervous system (CNS) [8]. For example, methamphetamine is a synthetic illicit psychomotor stimulant affecting the mesolimbic dopamine pathway, which plays an essential role in motivation and the reward system. A substantial body of evidence shows that acute methamphetamine use is associated with enhanced positive sexual experiences and libido, with a greater likelihood of engaging in high-risk sexual behaviors. Sexual behavior, however, may be impaired due to chronic methamphetamine exposure [9].

The modulation of neurotransmission is also involved in the mechanisms underlying many psychiatric and neurological disorders. For instance, although not fully understood, the dysregulation of monoaminergic systems, especially the decrease in serotoninergic, dopaminergic, and adrenergic neurotransmissions, seems to be the primary cause of depression [10]. Contrariwise, mania is believed to be the consequence of an excess of the same monoamines in specific regions of the brain [11]. Schizophrenia, however, mainly involves dopaminergic and glutamatergic neurotransmissions [12]. In neurology, a deficit of dopamine due to the death of cells in the substantia nigra is the primary cause of Parkinson’s disease, while Alzheimer’s disease is due to the degeneration of the hippocampus and the raphe nuclei, affecting the cholinergic pathway [13]. The drugs approved to treat psychiatric and neurological conditions mainly target these neurotransmission pathways.

Considering the mechanism of action of sexual enhancers, psychiatric and neurological adverse effects associated with their consumption are strongly expected. In 2014, Corazza et al. reviewed the psychoactive effects of four popular sexual enhancers [6]. In March 2020, Srivatsav et al. reviewed the efficacy and the safety profile of several common aphrodisiacs used in the treatment of erectile dysfunction, but the study was not exhaustive and psychiatric and neurological adverse effects were not addressed [14]. In this review, we aimed to summarize and interpret the findings of cases and preclinical and clinical studies reporting psychiatric and neurological adverse effects associated with the use of aphrodisiacs of plant origin. A preliminary screening of the literature allowed for the identification of several plants with potential psychiatric or neurological complications, which were therefore included in this review: *Areca catechu* L., *Argemone Mexicana* L., *Citrus aurantium* L., *Eurycoma longifolia* Jack., *Lepidium meyenii* Walp., *Mitragyna speciosa* Korth., *Panax ginseng* C. A. Mey, *Panax quinquefolius* L., *Pausinystalia johimbe* (K. Schum.) Pierre ex Beille, *Piper methysticum* G. Forst., *Ptychopetalum olacoides* Benth., *Sceletium tortuosum* (L.) N. E. Brown, *Turnera diffusa* Willd. ex. Schult., *Voacanga africana* Stapf ex Scott-Elliot, and *Withania somnifera* (L.) Dunal.

## 2. Results

**Figure 1:**
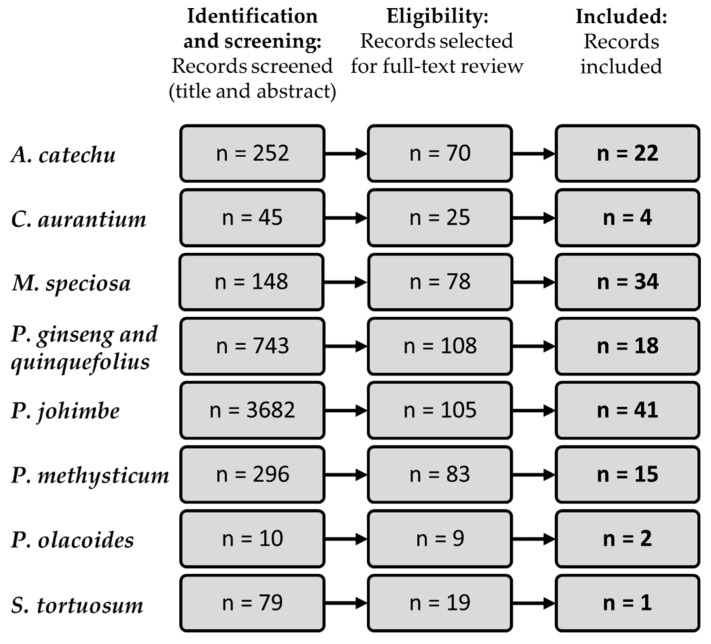
Species-by-species flow diagram of literature search.

Of 5255 potentially relevant reports, 4758 were excluded because they did not describe psychiatric or neurological adverse effects or because they were not written in English, French, or Italian language. No relevant reports were found for *A. Mexicana*, *E. longifolia*, *L. meyenii*, *T. diffusa*, *V. africana*, and *W. somnifera*, which were therefore excluded from the results. A total of 137 records were included in the final review; species-by-species search results are detailed in a flow diagram in Figure 1. A general description of each species including their traditional and modern uses, active ingredients with their mechanism of action and pharmacokinetics, and general toxicity, is provided in the discussion. Psychiatric and neurological adverse events reported in the literature are displayed in Table 1; study conditions and co-exposures are also detailed.

## 3. Discussion

### 3.1. Areca catechu L. (Betel Nut)

Betel nut is one of the most widely used addictive substances in the world and represents the fourth most consumed drug after nicotine, ethanol, and caffeine [23,154]. The fruit, obtained from the palm tree, *Areca catechu* L., is commonly chewed in Southeast Asia and the South Pacific islands [155,156] for its antiparasitic, digestive, euphoric, and aphrodisiac effects [157,158]. There are two main ways to prepare areca nut for chewing: wrapping a split unripe nut with lime paste (calcium oxide) in a betel tree leaf or inserting a piece of *Piper betel* L. inflorescence with lime paste into an unripe areca nut [22,154,159].

The betel nut contains a variety of active alkaloids responsible for its effects. The content of major alkaloids arecoline, arecaidine, guvacoline, and guvacine in the fresh nut is approximately 0.30–0.63%, 0.31–0.66%, 0.03–0.06%, and 0.19–0.72%, respectively, although it may vary with maturation [160,161,162,163]. Due to the presence of calcium oxide, arecoline and guvacoline are hydrolyzed to arecaidine and guvacine, respectively, in basic conditions [163,164]. Areca alkaloids mediate the autonomic responses of the parasympathetic nervous system and the synaptic transmission in the peripheral nervous system [164,165,166], and impact various aspects of brain function and regulation [167,168]. Arecoline is a partial muscarinic (M) agonist and possesses a higher affinity for M receptors than guvacoline [24,169]. High arecoline doses produce nicotinic cholinergic effects [170]. As tertiary amines, arecoline and, to a lesser extent, arecaidine have a deep brain penetration [165,169]. It was demonstrated that arecoline enhances cognition and memory and significantly improves several behavioral disorders in patients with Alzheimer’s disease or schizophrenia, through the activation of postsynaptic M1 receptors [24,162,171]. In addition, improvements in positive and negative symptoms of psychosis have been observed in schizophrenic areca nut chewers [155,172,173]. It is believed that arecoline reduces the dopaminergic hyperactivity underlying the positive symptoms of psychosis throughout the modulation of M1, M2, and M4 receptors, and induces dopamine release in the prefrontal cortex, the striatum, and the ventral tegmental area, ameliorating the negative symptoms [169,174,175]. Furthermore, the release of dopamine and other catecholamines causes stimulant and libido-enhancing effects [3,156,166,176,177]. These effects, coupled with the inhibition of monoamine oxidase A (MAO-A) caused by aromatic phenolic compounds of the plant, may also explain the antidepressant properties of the areca nut [24,169].

High betel nut doses can cause typical muscarinic and extrapyramidal symptoms such as salivation, diaphoresis, diarrhea, gastrointestinal upset, emesis, vertigo, myosis, tremor, hyperthermia, bradycardia, and asthma attacks [156,158,166,168,176,178,179]. Furthermore, if betel nut is taken in high quantities, transient extrapyramidal symptoms including rigidity, bradykinesia, and jaw tremor may occur [24,176,180]. These effects are probably due to guvacine and arecaidine, which are strong inhibitors of γ-aminobutyric acid (GABA) reuptake and contribute to the reduction in spontaneous activity and body excitability [181,182,183]. However, it was also demonstrated that areca alkaloids reduce GABA affinity for the GABA_A_ receptor, and arecaidine and guvacine effects on GABA signaling may cause epileptic seizures [23,169,180,184]. The main risks associated with the chronic consumption of betel nut are related to its metabolism. Indeed, areca alkaloids are converted into DNA alkylating nitrosamines, which cause cell proliferation and oxidative stress-dependent neurotoxicity, leading to oral carcinogenicity and exacerbating neurodegenerative disease symptoms [24,158,183,185]. Albeit its use is culturally well accepted, the betel nut is a strongly addictive substance and multiple adverse effects were reported (Table 1). Tolerance and nicotine/amphetamine-like withdrawal syndrome, characterized by insomnia, mood swings, irritability, and anxiety, may appear after repeated intakes [16,167,179].

### 3.2. Citrus aurantium L. (Bitter Orange)

*Citrus aurantium* L., also called Seville orange, sour orange, or bitter orange, is a small tree belonging to the *Rutaceae* family. It is native to Eastern Africa, Arabia, and Syria, but is also cultivated in Spain, Italy, and North America [186]. Bitter orange tree’s leaves, flowers, fruits peels, and seeds have been used for centuries to treat tachycardia, rheumatism, insomnia, anxiety, epilepsy, and gastrointestinal disorders and to enhance sexual desire [187,188,189].

The bitter orange tree contains vitamins, minerals, terpenoids, and flavonoids [190]. The most abundant flavonoids are hesperetin and naringenin, which possess, together with terpenoids, carotenoids, and ascorbic acid, a free radical-scavenging ability and inhibit proinflammatory mediators release, exerting a powerful antioxidant activity and reducing tumoral cell proliferation [191,192]. Terpenoids such as *d*-limonene, α-pinene, β-myrcene, linalyl acetate, and linalool are the main volatile components of the plant [193,194], and are distilled or extracted from blooms, leaves, and orange peel to obtain essential oils (EOs) [195]. These substances, especially d-limonene, modify the cell membrane of microbes and denature enzymes responsible for their germination and sporulation, showing antimicrobic and antifungal properties [191,196]. For these reasons, bitter orange tree’s EOs are widely sold as flavoring and preservative agents in foods and drinks [190,197]. Recently, a great interest in these EOs has been observed in the context of alternative medicines such as aromatherapy. It was demonstrated that these substances, after vaporization and inhalation, are effective for treating different forms of anxiety, sleep, and libido, and reduce seizures in animal models [38,39,198,199,200,201]. The mechanism of action of the plant’s ingredients, however, is not fully elucidated. Anxiolytic and hypnotic effects are seemingly due to the combination of the action of the plant’s aroma on the limbic system through olfaction and the direct action of terpenes on GABAergic and serotoninergic (5-HT_1A_) receptors [39,202,203].

Adverse effects are mild and transient; psychiatric and neurological effects are reported in Table 1. The US Food and Drug Administration confirmed that oral administration of bitter orange extracts is safe [39], and its derivatives have gained popularity as dietary supplements over the last few years [200,204]. Standardized derivatives containing 4–6% synephrine, an ephedrine-like alkaloid naturally occurring in *C. aurantium* and possessing α- and β-adrenergic properties, are available [3,40,205]. Although synephrine has become one of the most popular stimulants in weight loss products, cardiovascular adverse effects including increased blood pressure, tachycardia, ventricular fibrillation, transient collapse, myocardial infarction, and cardiac arrest have been reported [206]. Headache and gastrointestinal symptoms have also been reported.

### 3.3. Mitragyna speciosa Korth. (Kratom)

Kratom is an herbal preparation obtained from the leaves of *Mitragyna speciosa* Korth, an evergreen plant of the *Rubiaceae* family that grows spontaneously in Southeast Asia, mainly in Thailand and Malaysia [207]. In these countries, the plant has been exploited for centuries for its stimulant and narcotic properties and is commonly self-administrated by manual laborers to combat fatigue and improve productivity [51]. Mitragyna leaves are traditionally chewed, smoked, or boiled with hot water and served as a tea [208]. New preparations such as capsules, resins, or tinctures are now available on the Internet and are purchased in Europe and in America for recreational use or as herbal products [209,210].

More than 40 alkaloids were isolated from kratom. The alkaloids’ content is variable and depends on plant age, season, and geographical location [211]. Mitragynine is the most abundant active compound and accounts for up to 66% of the total mass of crude alkaloids extract. Other major alkaloids are paynantheine, which is the second most abundant alkaloid (10% of total content), speciogynine, and speciociliatine. Among the various minor alkaloids, 7-hydroxymitragynine is of particular interest due to its important role in mediating the analgesic effect of mitragynine [157]. Both mitragynine and its oxidized metabolite 7-hydroxymitragynine are partial agonists of µ-opioid receptors (MOR) and competitive antagonists of κ- and δ-opioid receptors (KOR and DOR), which are involved in analgesia. However, mitragynine affinity for opioid receptors is lower than that of morphine, while 7-hydroxymitragynine affinity is approximately 46 and 13 times higher than that of mitragynine and morphine, respectively [212]. The possible stimulant and libido-enhancing effects of mitragynine may be due to the blockade of serotonergic 5-HT_2A_ receptors and the postsynaptic stimulation of α_2_ adrenergic receptors (α_2_R) in the CNS [212]. Recently, LaBryer et al. hypothesized that kratom may have restored testosterone, luteinizing hormone (LH), follicle-stimulating hormone (FSH), and prolactin levels in a patient with hypogonadotropic hypogonadism [213]. Mitragynine also binds adenosine A_2A_ receptors, dopamine D_2_ receptors, and serotonin receptors 5-HT_2C_ and 5-HT_7_, but the physiological significance of these interactions is unclear [214]. Data showed that low kratom doses (1–5 g) induce stimulating effects, involving the release of neurotransmitters by reversible blockade of calcium channels. At higher doses (>15 g), it can cause sedative/narcotic effects, and the plant can be used as a general analgesic, as an opium substitute, or to treat opium withdrawal symptoms [215].

Studies have found that 7-hydroxymitragynine is the main contributor to the plant’s toxicity and the development of addiction symptoms. In 2014, Singh et al. [51] reported a cross-sectional survey investigating the correlation between frequency and quantity of kratom consumption, and the risk of addiction development and the severity of withdrawal symptoms and craving in regular user [216]. Cessation of kratom use produced physical withdrawal symptoms similar to those of opiate addiction including pain, sleep disorders, muscle spasms, watery eyes, runny nose, hot flashes, fever, decreased appetite, diarrhea, and craving. Psychological withdrawal symptoms reported by users included restlessness, tension, anger, and depression [217] (Table 1).

### 3.4. Panax ginseng C. A. Mey (Asian Ginseng) and Panax quinquefolius L. (American Ginseng)

Ginseng is a perennial herbaceous plant of the family *Araliaceae*. Over twelve ginseng species were identified, although mainly Asian or Korean ginseng (*P. ginseng*) and American ginseng (*P. quinquefolius*) have been used for their therapeutic properties. Asian ginseng grows in East Asian mountains, while American ginseng is an endangered species that grows in deciduous forests of the west half of North America. Asian ginseng is an emblematic plant of traditional Chinese medicine, and is listed in the *Shennong Ben Cao Jing*, the most ancient Chinese book of herbal medications. A variety of pharmacological effects are attributed to ginseng. For example, it is used to improve intelligence, to treat impotence, to treat hemorrhage, to relax, and to slow aging [218]. American ginseng medical use is more recent and has gained popularity in Western countries over recent decades. Ginseng health benefits were demonstrated in many clinical studies in various fields: it has shown aphrodisiac, anti-inflammatory, anticancer (lung, liver, intestine, and stomach), antidiabetic, cardioprotective, gastroprotective, antiamnestic, and antioxidative (heart and kidney) properties [219]. The fresh root of ginseng can be directly chewed after peeling or soaked in wine for drinking and chewing. In China and Korea, it is boiled with chicken to prepare energy drinks, teas, and candies [218].

Asian and American ginseng contain a variety of pharmacologically active triterpene saponins, named ginsenosides. Ginsenosides are classified into two groups depending on the hydroxylation of their steroid core structure: the 20(*S*)-protopanaxadiol (PPD) and 20(*S*)-protopanaxatriol (PPT) groups. Rb1, Rb2, Rb3, Rc, and Rd are the main PPD-type ginsenosides, and Rg1 and Re are the main PPT-type ginsenosides. A total of 32 ginsenosides, including the abovementioned compounds, are found in in both American and Asian ginseng, but the two plants also possess specific ginsenosides. The presence of Rf, a PPD/PPT ratio lower than 2, and a Rb1/Rg1 ratio lower than 5 usually identify Asian ginseng. The content of ginsenosides is affected by seasons, geographical distribution, and processing (fresh ginseng, steamed ginseng or white ginseng, and sun-dried ginseng or red ginseng) [220]. After oral administration, ginsenosides are mainly metabolized in the gastrointestinal tract and the liver, undergoing successive deglycosylations: Rg3 is a metabolite of Rb1, Rb2, Rb3, Rc, and Rd, and is further metabolized to Rh2; Rg2 is a metabolite of Re; and Rh1 is a metabolite of both Rg1 and Rg2. Ginsenosides have low oral bioavailability, owing to their low membrane permeability and their degradation in the gastrointestinal tract [219]. Rg1 induces NO synthesis in endothelial cells and perivascular nerves, and increases vascular smooth muscle sensitivity to NO, prolonging the erection in males and enhancing sexual potency. In addition, *P. ginseng* was proved to increase testosterone, LH, and FSH in healthy volunteers through Rg1 and Rb1, enhancing libido. Re increases extracellular dopamine and acetylcholine in rat brains, while Rb1 increases choline reuptake at the synapses. Rb1, Rb2, Rc, Re, Rf, and Rg1 are agonists of GABA_A_ receptors and Rc is also an agonist of GABA_B_ receptors. These modulations of several neurotransmission pathways may have an effect at different levels of the hypothalamus–pituitary–testis axis [221]. Mancuso and Santangelo recently reviewed the effects of ginsenosides on the immune system (e.g., modulation of the immune response, anti-inflammatory effects), the nervous system (e.g., regulation of the stress axis, improvement of memory and learning functions), and the cardiovascular system (e.g., improvement of cardiac performance, cardioprotective effects) [222].

American and Asian ginseng present a good safety profile with a few cases of mild gastrointestinal and sleep disorders. Psychiatric and neurological side effects are rare, and causality is difficult to ascertain [77,79,80,82,83,84,85,86,87,89,90,91,92,93,94]. In fact, most clinical studies with ginseng or ginsenosides reported no psychiatric or neurological side effects or statistically insignificant effects compared to placebo, and were not included in Table 1 [223,224,225,226,227]. A few cases of headache following ginseng administration were reported without placebo control [84,91]. Insomnia, agitation, and fatigue were more frequent [82,83,84,89,94], but still uncommon. More interestingly, several cases of manic-like effects, such as confusion, agitation, irritation, nervousness, anxiety, and bizarre behavior, without history of psychiatric disorder, were reported following ginseng use [77,78,81,85,87,88,90,228]. These cases, however, were isolated and generally resulted from high ginseng doses or with the concomitant use of other substances (e.g., phenelzine, cannabis, herbal supplements); mania was not reported in clinical studies using ginseng alone or in combination. In fact, ginseng toxicity mainly comes from drug interactions with cytochromes P450 (CYPs) CYP3A4 and CYP2D6 inhibitors and serotoninergic drugs, intensifying sedative effects or inducing cognitive disorders or a serotonin syndrome [229,230,231].

### 3.5. Pausinystalia johimbe (K. Schum.) Pierre ex Beille (Yohimbe)

*Pausinystalia johimbe* (K. Schum.) Pierre ex Beille, also known as yohimbe, is an evergreen tree of the *Rubiaceae* family that mainly grows in the tropical region of the African West coast, where the bark has been consumed as an aphrodisiac for the treatment of erectile dysfunction [157,232].

The bark of the plant contains several structurally related indole alkaloids, yohimbine being the most abundant one (10–15% of total content), followed by its stereoisomers α-yohimbine, β-yohimbine, ψ-yohimbine, corynanthine, *allo*-yohimbine, and yohimbic acid [233,234]. Yohimbine is marketed as a pharmaceutical prescribed for the treatment of erectile impotence and has been used in multiple clinical trials as a probe to identify abnormal physiological and affective responses to increased noradrenergic signaling, especially in patients with panic disorders [235,236]. Yohimbine is a potent selective α_2_R antagonist with weaker α_1_R antagonist activity that blocks the presynaptic feedback inhibition of noradrenaline release, prolonging the excitatory effects of noradrenaline at postsynaptic α_1_R and β-receptors [237,238]. Yohimbine has a relatively short half-life due to an extensive hepatic metabolism, which produces two main hydroxylated metabolites, 11- and 10-hydroxyyohimbine, that are rapidly excreted in urine [239,240]. There is increasing interest in botanic dietary supplements containing yohimbine extracts in the context of sexual and body enhancement [241].

Yohimbine readily passes the blood–brain barrier after absorption, causing an increase in sympathetic tone and blood pressure through the blockade of central medullary α_2_R [242], and provokes noradrenergic perturbation in limbic forebrain structures like amygdala and locus coeruleus, leading to mood and behavior alterations (Table 1). Due to these potential cardiac and neurological adverse effects, yohimbine was used in patients with difficulties to reach orgasm, with erectile dysfunction, or with low libido, mainly before the emergence of phosphodiesterase 5 (PDE5) inhibitors (e.g., sildenafil), which present a better safety profile [243,244]. The yohimbine mechanism of action is currently unclear. There is evidence suggesting that yohimbine increases libido by blocking α_2_R in the locus coeruleus, which is involved in the control of the erection. Peripherally, it was suggested that yohimbine enhances NO release from cavernosal endothelial cells, producing a relaxation of smooth muscle cells and consequent erection, increasing sexual potency [3,6,237]. Moderate to severe adverse effects like sweating, flushing, hypertension, tachycardia, palpitations, bronchospasm, chest pain, and atrial fibrillation are reported after deliberate or accidental ingestion, while lethal intoxications are extremely rare [245,246].

### 3.6. Piper methysticum G. Forst. (Kava)

Native to Western Pacific islands where the shrub is traditionally called “ava”, “wati”, or “yagona”, kava (*Piper methysticum* G. Forst) grows in humid and shaded areas of tropical regions. This is a perennial *Piperaceae* with a massive rhizome weighing up to a dozen kilograms [19]. Kava is considered as a sacred beverage in Pacific islands [247]. The plant has also been used in traditional medicine, first as a treatment for venereal diseases, then later as a sedative and treatment for anxiety and sleep disorders, to decrease fatigue, and to relieve pain [248].

Eighteen active compounds named kavalactones were identified in kava, but only six, i.e., methysticin, dihydromethysticin, kawain, dihydrokawain, desmethoxyyangonin, and yangonin, have been the focus of kava studies as they account for up to 96% of organic extracts (acetonic or ethanolic extraction). Kava also contains a variety of other non-lactone compounds, i.e., flavokawains A, B, and C, 5,7-dimethoxyflavanone, cinnamic acid bornyl ester, flavanones, fatty acids, and a chalcone. Kava effects may be the result of a synergy of the six major kavalactones [249]. Kava extracts produce a similar activity profile as that of benzodiazepines, which interact with GABA receptors, inhibit the MAO-B, and inhibit dopamine and noradrenaline reuptake in the CNS, inducing libido-enhancing properties [250]. In vitro studies of the hippocampus and other brain regions suggest that the sedative effects of kavalactones may be mediated by an increase in GABA_A_ receptor binding sites [251,252]. It was demonstrated that several kavalactones are potent inhibitors of several CYP metabolic enzymes [253]. Oral pharmacokinetics of kawain (100 mg/kg) were determined in rats with and without co-administration of kava extract (256 mg/kg). The results showed that kawain was well absorbed, and more than 90% of the dose was eliminated within 72 h, mainly in urine [254].

Data on kava’s safety profile obtained from clinical trials in patients with anxiety suggest generally good tolerability and safety for short-term use (1–4 weeks) at therapeutic doses. However, the use of kava at frequent and high doses can cause hepatotoxicity through the modulation of various CYP, which is also the cause of potential drug interactions [255], dermopathy [256], and cognitive disorders [257]. Sedation with drowsiness and dizziness is commonly reported in clinical trials with administration of kava or methysticin (Table 1), which may impair driving performances [148].

### 3.7. Ptychopetalum olacoides Benth. (Muirapuama)

*Ptychopetalum olacoides* Benth. is a popular Amazonian tree belonging to the *Olacaceae* family. It is also known in Brazil as *marapuama* or *muirapuama* [258]. The native communities have used its roots and barks as a treatment for depression, sexual dysfunction, and as a “nerve tonic” [259]. Roots are usually prepared in alcoholic infusion, but other formulations have also been employed (e.g., mixture of extracts, solutions, pills) [260].

Muirapuama root bark produces a volatile oil containing α-pinene, α-humulene, β-pinene, β-caryophyllene, camphene, and camphor. Fatty acids such as uncosanoic, tricosanoic, and pentacosanoic acids account for up to 20% of the total lipophilic components of the plant [261]. Other compounds detected in muirapuama are fatty acid esters of sterols, coumarin, free fatty acids, and free sterol; a small quantity of β-sitosterol was also detected [262]. Based on ethnopharmacological data, it can be hypothesized that muirapuama interacts with the dopaminergic system, increasing libido; the noradrenergic system, inducing antidepressant effects; and the serotonergic system, modulating appetite. The antinociceptive effects of the plant were investigated in thermal and chemical models of nociception in mice. Data showed that the maximal effect was reached 6 h after administration of a low dose of muirapuama extract and produces significant and long-lasting (up 12 h) effects in both chemical and thermal tests of nociception in mice. In addition, higher doses, either acutely or subchronically (15 days), did not cause a worsening of adverse effects [263]. A few neurological side effects were reported in preclinical studies, including impairment of both short- and long-term memory and reduced locomotion [151,152] (Table 1).

### 3.8. Sceletium tortuosum (L.) N. E. Brown (Kanna)

Kanna, or Channa, is the traditional name of a succulent, perennial plant belonging to the *Aizoaceae* family (earlier *Mesembrynantheaceae*) that is indigenous to South Africa, where it is consumed by local tribes to relieve thirst and hunger and combat fatigue [264,265]. The dried aerial parts of the plant are commonly chewed or consumed as teas, decoctions, and tinctures and sometimes smoked or snuffed [266].

Kanna’s main active compounds are mesembrine, mesembrenol, mesembrenol, and mesembrenone, which are synthesized in the plant through the condensation of phenylalanine and tyrosine amino acids. Kanna alkaloids inhibit serotonin reuptake and PDE4A, potentially enhancing sexual potency and libido [267,268,269]. In fact, the plant is widely sold over the Internet and specialized herbal shops to increase sexual performance, although its mechanism of action and effects were not clearly demonstrated. This dual inhibitory effect has been studied for the development of compounds for the treatment of cognition impairment, motor dysfunction, depression, and neurodegeneration [270]. Mesembrenone is the most potent inhibitor of PDE4A, while mesembrine is more selective towards the serotonin receptor. In addition, mesembrine is also an agonist of GABA_A_, δ_2_-opioid, μ-opioid, cholecystokinin-1, E4-prostaglandin, and melatonin-1 receptors at high doses, and shows antinociceptive effects in animal models [266,270,271,272,273]. Recently, *S. tortuosum* has been marketed for the treatment of mild depression and to improve mood [274]. Zembrin^®^, a standardized hydroalcoholic extract, was found to be safe and well-tolerated in preclinical studies with mild adverse effects (Table 1) and is widely sold as a dietary supplement [275]. Another commercially available product is Trimesemine™, a highly-concentrated mesembrine extract (3% mesembrine (*w*/*w*)) that shows a great upregulation of the expression of vesicular monoaminetransporter-2 (VMAT-2), increasing monoamines release as the primary pharmacological effect [276,277]. Kanna alkaloids are metabolized in the liver to *O*,*N*-demethylated or dehydroxylated compounds, which are then excreted as glucuronides or sulfates conjugates [277,278].

### 3.9. Other Plants

Little evidence was found on the psychiatric and neurological side effects of several sexual enhancers that were originally included in this review: *A. Mexicana*, *E. longifolia*, *L. meyenii*, *T. diffusa*, *V. africana*, and *W. somnifera*.

The safety of maca (*Lepidium meyenii* Walp.) and ashwagandha or Indian ginseng (*Withania somnifera* (L.) Dunal) is well-documented. Maca is an edible herbaceous plant of the Andes Mountains and has been used for centuries for improving male and female sexual function. A renewed interest for maca has been observed from the 1990s onwards, and pills, capsules, flour, liquor, and extracts are now massively produced and exported [279]. The plant, however, presents a good safety profile [279], and only one case of a manic-like episode following consumption in a patient without history of psychiatric disorders was reported [280]. Ashwagandha is an evergreen shrub of the family *Solanaceae*, which is cultivated in hot and dry areas of tropical and subtropical regions of the world. Ashwagandha has numerous applications in traditional Indian medicines and has been used for more than 5000 years as an aphrodisiac, antioxidant, antimicrobial, adaptogenic, diuretic, tonic, narcotic, immuno-stimulant, anti-inflammatory agent, anti-stress, antiulcer, among many other purposes. Positive effect in insomnia, anxiety, chronic stress, weight management, thyroid gland function, telomerase, cardio-respiratory endurance, muscle strength, recovery of male and female sexual function, and senescence was demonstrated in preclinical and clinical studies [164]. Excellent tolerability is generally reported after using ashwagandha for weeks at therapeutic doses, with statistically insignificant adverse effects compared to placebo [281,282].

Other plants have been little studied, although some of them are well-known remedies that have been used for many years. Further investigation is necessary to clearly understand the possible neurological and psychiatric effects caused by these plants. For example, damiana (*Turnera diffusa* Willd. Ex. Schult.), a Central American shrub of the family *Passifloraceae*, has been traditionally used for centuries as a stimulant and aphrodisiac, to spark male sexual drive and increase performance, and is still widely marketed [283]. Recently, the plant also proved to induce anxiolytic and antidepressant effects in preclinical studies [283,284]. However, no psychiatric or neurological side effects related to damania were reported. Another interesting example is *Voacanga africana* Stapf ex Scott-Elliot, a shrub/small tree of tropical Africa whose seeds have been recently commercialized as a poison, stimulant, aphrodisiac, and ceremonial psychedelic [285]. Although the plant contains a variety of indole alkaloids related to ibogaine, a well-known psychoactive substance from iboga (*Tabernanthe iboga* Baill.) that has also been used as an aphrodisiac [286], there is no evidence of positive effects on sexual function. In addition, although *Voacanga africana* alkaloids may modulate neuronal excitability and have positive effects in Alzheimer’s disease [287,288], no neurological or psychiatric complications related to the plant were reported. There is also little information on the side effects of the Mexican poppy (*Argemone mexicana* L.) and Tongkat Ali (*Eurycoma longifolia* Jack.).

Other herbal aphrodisiacs that were not included in this literature review are commonly used, but data on their safety profile are also limited [14].

### 3.10. General Discussion

The psychiatric and neurological side effects caused by the consumption of herbal aphrodisiacs are mild, with mostly no consequences at therapeutic doses. Although rare, psychiatric side effects were mostly reported and included anxiety, depression, psychosis, and mania. Neurological effects included seizures, extrapyramidal symptoms, and withdrawal symptoms. Dizziness, sleepiness, fatigue, agitation, insomnia, and headaches were reported. As expected, all the plants involved are libido-enhancing aphrodisiacs altering neurotransmission, although ginseng (*P. ginseng* and *P. quinquefolius*) and yohimbe (*P. johimbe*), and kanna (*S. tortuosum*) also enhance sexual potency. Kratom (*M. speciosa*) and yohimbe presented the highest rate of side effects, but yohimbe’s general toxicity is mainly cardiovascular, due to its effects on the NO pathway. These mild effects were not anticipated, considering the mechanism of action of these plants. However, they did not come as a complete surprise, because these plants have been used for centuries or millennia in traditional medicines and modern medicine has considerably benefited from these ingredients and preparations [289].

Few clinical reports and few data were found for the side effects of bitter orange (*C. aurantium*), muirapuama (*P. olacoides*), and kanna (*S. tortuosum*), and only preclinical reports were found for muirapuama. There were no reports on the side effects of damania (*T. diffusa*), the Mexican poppy (*A. mexicana*), and Tongkat Ali (*E. longifolia*). Although several of these plants have been used for centuries in traditional medicines, they have not been as extensively studied as the other plants included in this review. Therefore, this result was not surprising. Psychiatric and neurological side effects included headaches and hypnotic effects and were mild. Although the consumption of these plants appears safe, further studies should be conducted to better understand their positive and negative effects.

Herbal aphrodisiacs are readily available on the Internet and specialized shops and markets. Aside from sexual enhancement, these plants often have many other therapeutic applications and can be a part of the everyday diet of individuals. In addition, self-medication is frequent, as they are available without a prescription, and plant extracts provided by retail herbal stores may also be mislabeled or adulterated (e.g., heavy metals, pharmaceuticals) [290]. Consequently, these plants are often taken concomitantly with contaminants, medications, and other herbal therapies or dietary supplements: drug interactions represent the most significant health risks of herbal aphrodisiacs and psychiatric and neurological adverse effects have been reported [230]. Medical doctors should be aware of the potential drug interactions with aphrodisiacs and dietary supplements when prescribing a pharmaceutical drug.

### 3.11. Limitations

Considering the paucity of data and the frequent co-exposure to other psychoactive substances, conclusions can be hardly drawn regarding the psychiatric/neurological adverse effects of several of the plants included in this article. In particular, more clinical studies with controlled administrations, to have a better grasp on doses and co-exposures, should be conducted to address this issue.

Another limitation is the choice of the keywords used for the literature search. The review focused on selected plants and their active ingredients, which were included after a preliminary screening of the literature. Other less common herbal aphrodisiacs might have eluded the search.

Additionally, only articles written in English, Italian, and French were included in the review, and reports written in another language were excluded. This limitation is particularly relevant considering the prevalence of traditional Ayurvedic, Unani, and Chinese medicines in East Asia and diasporas [291].

## 4. Materials and Methods

A comprehensive literature search was conducted using the PubMed, Scopus, and Web of Science bibliographic databases to identify scientific reports on the psychiatric and neurological complications associated with the use of *A. catechu*, *A. mexicana*, *C. aurantium*, *E. longifolia*, *L. meyenii*, *M. speciosa*, *P. ginseng*, *P. quinquefolius*, *P. johimbe*, *P. methysticum*, *P. olacoides*, *S. tortuosum*, *T. diffusa*, *V. africana*, and *W. somnifera*. Database-specific search features with truncations (represented by an asterisk in this article) and multiple keywords (represented by quotation marks in this article) were employed. The search terms employed were addict*, anxiety, anxious, “brain disorder *”, cognit *, depression, depressive, hallucin *, insomnia *, mania *, manic, mental, panic, “personality disorder *”, psychiatry *, psychosis, psychotic, or schizoph * in combination with the following terms for each plant:

- *A. catechu*: “Areca catechu”, “areca palm”, “areca nut palm”, “betel palm”, “Indian nut”, “Pinang palm”, arecaidine, or arecoline;
- *A. Mexicana*: “Argemone mexicana”, “Mexican poppy”, “flowering thistle”, sanguinarine, dihydrosanguinarine, dehydrocorydalmine, jatrorrhizine, columbamine, or oxyberberine;
- *C. aurantium*: “Citrus aurantium”, “bitter orange”, “Seville orange”, “bigarade orange”, or “marmalade orange”;
- *E. longifolia*: “Eurycoma longifolia”, “tongkat ali”, “pasak bumi”, “Malaysian ginseng”, eurycomanol, eurycomanone, or eurycomalactone;
- *L. meyenii*: “Lepidium meyenii”, maca, or “Peruvian ginseng”;
- *M. speciosa*: “Mitragyna speciose”, kratom, biak, mitragynine, or hydroxymitragynine;
- *P. ginseng* and *P. quinquefolius*: “Panax ginseng”, “Panax quinquefolius”, ginseng, or ginsenoside*;
- *P. johimbe*: “Pausinystalia johimbe”, yohimbe, ajmalicine, allo-yohimbine, corynantheine, pseudoyohimbine, raubasine, yohimbine;
- *P. olacoides*: “Ptychopetalum olacoides”, “muira puama”, or muirapuamine;
- *S. tortuosum*: “Sceletium tortuosum”, kanna, channa, kougoed, mesembrine, mesembrenone, mesembrenol, or tortuosamine;
- *T. diffusa*: “Turnera diffusa”, damania, or damianin;
- *V. Africana*: “Voacanga africana”, voacamine, or voacangine;
- *W. somnifera*: “Withania somnifera”, ashwagandha, “Indian ginseng”, or “poison gooseberry”.

Further studies were retrieved from the reference list of selected articles and reports from international institutions such as the World Health Organization (WHO), the US Drug Enforcement Administration (DEA), and the European Monitoring Centre for Drugs and Drug Addiction (EMCDDA).

Records reporting the psychiatric and/or neurological adverse effects associated with the use of the selected plant species in humans were included; only articles written in English, French, and Italian were included. Databases were screened up until June 2020 and references were independently reviewed by three of the authors to determine their relevance to the present article.

## 5. Conclusions

Most of the sexual enhancers of plant origin included in this review appeared to be safe at therapeutic doses, and few psychiatric and neurological side effects were reported. Yohimbe mainly was involved in intoxication cases, but its cardiovascular toxicity is more concerning than its psychiatric and neurological adverse effects. In addition, causality was often difficult to determine, especially in case reports and self-reports, in which co-administration of other substances and herbal formulations is prevalent. Interactions with pharmaceuticals or other herbal supplements are more common and may pose a significant health threat.

The mechanisms underlying psychiatric and neurological disorders are multifaceted, often involving multiple CNS pathways and receptors, and are not fully understood. Similarly, herbal aphrodisiacs often contain multiple active ingredients with multiple mechanisms of action that are yet to be fully characterized. Consequently, the mechanisms underlying the psychiatric and neurological side effects associated with herbal aphrodisiac use is little-known.

Other sources of aphrodisiacs affecting the CNS are available, such as synthetic or semi-synthetic drugs and natural substances of animal origin, which may also induce psychiatric or neurological effects. Although aphrodisiacs are mainly obtained from plants, these substances may also be worthy of investigation. It is also important to consider that plants that do not induce psychiatric or neurological adverse effects are not necessarily harmless, and could exhibit another type of toxicity (e.g., cardiovascular, hepatic, and renal toxicity), which was not the focus of the present review.

## Figures and Tables

**Table 1:** Reports of psychiatric and neurological adverse events associated with herbal aphrodisiacs.

Plant/Active Ingredients	Co-Exposure	Study, Participants, Age, and Sex	Neurological/Psychiatric Effects	References
*Areca catechu* L.	Fluphenazine decanoate, procyclidine, flupentixol	Case reports of acute intoxications with *Areca catechu* L. in two schizophrenic men aged 51 and 45; both patients consumed a high quantity of betel nuts for 2 weeks	Rigidity, bradykinesia, akathisia, and tremors	[15]
	Tobacco, alcohol, cannabis, amphetamine	Survey study on 11 participants aged 27–70 (average = 52), 9 males and 2 females; chewed 6 nuts/week (range = 4–6 days)	Mood swings, anxiety, irritability, reduced concentration, reduced energy, sleep disturbance, craving, tolerance, and dependence	[16]
	Tobacco, salbutamol, tea, coffee, arsenic	Longitudinal pilot study on 100 participants, 26 users with a mean (±SD) age of 40.0 (±9) years, 11 males and 15 women	Enhancement of physiological tremor	[17]
	Tobacco, alcohol	Cross-sectional study on 310 pregnant women, 292 users, mean age of 26 years (range = 25–27); took 5–10 nuts during pregnancy	Addiction	[18]
	Tobacco	Survey study on 59 participants, 47 males and 12 females, median age of 43.0 years (range = 12–70); 1–50 years of chewing	Craving and dependence	[19]
	Tobacco	Cross-sectional study on 851 participants, aged 16–35, 314 users, 242 tobacco + users, and 295 regular cigarette smokers; <6–>10 years of chewing	Tolerance and withdrawal	[20]
	Tobacco, alcohol	Intercountry Asian BQ Consortium study on 2078 participants who took any *A. catechu* products/day for a minimum of 6 months	Tolerance, withdrawal, craving, and dependence syndrome	[21]
	Tobacco, alcohol	Survey study on 41 participants with a mean (±SD) age of 40.34 (±9.23) years, 27 males and 14 females; took 5 to more than 31 BQ/day	Relaxation, stimulation, addiction, and withdrawal symptoms	[22]
	Benzodiazepines, carbamazepine, levetiracetam, phenobarbital, phenytoin, sodium valproate, other medications (not specified)	Observational study on 152 participants with epilepsy, 50 users with a mean age of 28.4 (95% CI: 25.3, 31.6) years, 23 males and 27 women; chewed 1–20 nuts/day for more than 5 years	Drowsiness	[23]
	Tobacco and alcohol-	Epidemiological studies of dependence on 4031 participants Case report of two women	Dependence, tolerance, withdrawal, attentional bias, impaired work, impaired time perception, and increased arousal Poor concentration, lethargy, despondency, and episodes of paranoia (1 individual)	[24]
	Tobacco	Survey study on 200 participants, 171 males and 29 females aged 22–45 years; chewed 4.3 nuts/day	Addiction	[25]
	Nicotine, arecaidine (1.46 µg/mL in urine), N-methylnipecotate, cotinine	Cross-sectional survey on 113 participants with a mean (±SD) age of 40.0 (±12.6) years, 104 males and 9 females	Craving for BQ, addiction, depression, and drowsiness	[26]
Arecoline	Methscopolamine	Cholinergic REM induction test on 34 participants: 14 bipolar patients with a mean (±SD) age of 30 (±6.1) years, 6 males and 8 females; 15 HC volunteers with a mean (±SD) age of 26.8 (±4.4) years, 8 males and 7 females; and 5 subjects with a personal or family history of affective disorders, age ranged between 23 and 35 years, 2 males and 3 females; administered with 0.5 mg of arecoline	Shorter REM latency in patients with primary affective disorders (1st and 3rd)	[27]
	Glycopyrrolate	Pilot dose–response study on 24 participants, 8 bipolar subjects and 16 HC monozygotic twins; administered with 1 or 12 mg of arecoline IP and 4 or 12 mg SC	Nausea, vomiting, increased anger, confusion, depression, fatigue and tension, decreased elation, friendliness, and vigor	[28]
	Glycopyrrolate	Arecoline REM induction test on 97 participants: 20 MDD patients with a mean age of 46.6 years (range 22–83), 8 males and 12 females; 19 MDD+ANX patients with a mean age of 40.0 years (range 21–47), 7 males and 12 females; 18 ANX patients with a mean age of 32.2 years (range 22–51), 8 males and 10 females; 14 ANX+MDD patients with a mean age of 32.8 years (range 23–38), 4 males and 10 females; and 26 healthy controls with a mean age of 36.4 years (range 20–87), 11 males and 15 females; administered with 0.5 mg of arecoline	Rapid REM induction in MDD and MDD+ANX patients compared to healthy controls	[29]
	Methyl atropine	Animal study on 265 Sprague Dawley rats; administered with 2 mg/kg of arecoline	Decreased locomotory activity	[30]
	Glycopyrrolate	Cognitive and behavioral study on 12 participants with AD, mean age of 65.5 years (range 54–79), 4 males and 8 females; administered with 0.5 and 12 mg/h of arecoline for 1 to 6 h	Decreased knowledge memory, psychomotor retardation, dysphoria, and difficulty with verbal expression	[31]
	Glycopyrrolate	Arecoline REM induction test on 30 participants: 10 with atypical depression with a mean (±SD) age of 33.5 (±7.8) years, 4 males and 6 females, and 20 HC with a mean (±SD) age of 34.5 (±13.8) years, 10 males and 10 females; administered with 0.5 mg of arecoline	Rapid REM induction in atypical depressives without a history of panic attacks or anxiety disorders compared to HC	[32]
	Glycopyrrolate	Catecholamine and ACTH responses to an arecoline study on 31 participants: 15 MSA patients with a mean (±SD) age of 57.9 (±1.8) years, 8 males and 7 females; 6 PAF patients with a mean (±SD) age of 52.3 (±1.8) years, 2 males and 4 females; and 10 HC with a mean (±SD) age of 58.5 (±4) years, 7 males and 3 females; administered with 0.3 mg of arecoline	Mild vertigo, nystagmus, nausea, exacerbation of tremor, and alteration in mood	[33]
	Glycopyrrolate	Randomized, double-blind, placebo-controlled study on 111 participants: 40 placebo-receiving subjects, including 20 depressed patients with a mean (±SD) age of 39 (±12) years and 20 HC with a mean (±SD) age of 28 (±6) years; 38 subjects receiving 0.5 mg of arecoline, including 21 depressed patients with a mean (±SD) age of 38 (±9) years and 17 HC with a mean (±SD) age of 28 (±6) years; and 33 subjects receiving 1.0 mg of arecoline, including 18 depressed patients with a mean (±SD) age of 42 (±11) years and 15 HC with a mean (±SD) age of 34.5 (±13.8) years	Shorter REM latency in depressed patients	[34]
	-	Cholinergic REM induction test on 48 participants: 33 MDD children with a mean (±SD) age of 10.5 (±1.5) years, 26 males and 7 females and 15 HC children with a mean (±SD) age of 10.2 (±1.6) years; administered with 0.5 mg of arecoline over 60 min	Shorter REM latency in depressed children	[35]
	-	Animal study on 63 male albino Wistar-derived rats; administered with 0.5, 1.5, or 3.5 mg/kg of arecoline SC before test	Accelerated decay of memory after chronic administration	[36]
	-	Animal study on 96 female Sprague Dawley rats; administered with 40 or 80 µg of arecoline	Decreased locomotory activity	[37]
*Citrus aurantium* L.	Chlordiazepoxide, valproic acid, diazepam, sodium pentobarbital, *d*-limonene	Animal study on 176 adult male Swiss mice; administered with 0.5 or 1.0 mg/kg of EO (90.4% of *d*-limonene) and 1.0 mg/kg of four different fractions of leaves extract orally	Increased hypnotic effect and enhanced sleeping time induced by pentobarbital	[38]
	-	Randomized, triple-blind, clinical study on 156 postmenopausal women: 52 CA-receiving women with a mean (±SD) age of 53.65 (±3.55) years, 52 lavender-receiving women with a mean (±SD) age of 54.21 (±3.86) years, and 52 placebo-receiving women with a mean (±SD) age of 52.12 (±3.49) years; took 2 × 500 mg/day of CA powder for 6 weeks	Headache, nausea, and hypnosis	[39]
	-	Randomized, triple-blind, clinical study on 156 postmenopausal women: 52 CA-receiving women with a mean (±SD) age of 53.65 (±3.55) years, 52 lavender-receiving women with a mean (±SD) age of 54.21 (±3.86) years, and 52 placebo-receiving women with a mean (±SD) age of 52.12 (±3.49) years; took 2 × 500 mg/day of CA powder for 6 weeks	Headache and nausea	[40]
	*Rosa damascena* Mill.	Randomized, double-blind clinical study on 99 female students with a mean (±SD) age of 22.33 (±2.38) years, equally parted into two intervention groups and a control group; inhaled EO of CA blossom at 0.5%	Headache, nausea, and vomiting	[41]
*Mitragyna speciosa* Korth.	-	Cross-sectional study on 433 participants with a mean (±SD) age of 45.7 (±13.6) years, 350 males and 83 females; 149 regular users chewed 12 leaves/day, 168 occasional users chewed 4 leaves/day, and 116 controls	Dizziness, freshness, sprightliness, fatigue, shaking hands, headaches, decreased sexual drive, poor concentration, distractedness, difficulty sleeping, irritability, poor thinking ability, impaired memory, laziness, and social withdrawal	[42]
	Modafinil	Case report of a kratom intoxication of a 43-year-old male with chronic pain and opioid withdrawal; took kratom tea four times daily for 3.5 years	Withdrawal, drowsiness, and generalized tonic-clonic seizure	[43]
	*Datura stramonium* L., cannabinoids, tricyclic antidepressants, oxycodone	Case report of a kratom intoxication of a 64-year-old male, found with 167 ± 15 ng/mL of mitragynine in urine	Seizures and coma	[44]
	heroin, cannabis, amphetamine, methamphetamine, mitragynine (67.5–75 mg/day)	Cross-sectional survey on 136 participants: 72 short-term users and 64 long-term users, age range = 36–65 years, 135 males and 1 female; took 3 × 250 mL/day of kratom tea for 3.5 years	Loss of weight, tiredness, dose-dependent stimulation/sedation, intense craving, withdrawal, chronic fatigue, insomnia, frequent sweating, and sudden nerve pain	[45]
	-	Case report of a 44-year-old kratom addicted male with a previous history of alcohol and cocaine dependence; took from 2 × 4 g/day to 4 × 10 g/day for 3 years	Wellbeing, euphoria, increased productivity, industriousness, relaxation, tolerance, withdrawal symptoms of cravings, anxiety, restlessness, and itch	[46]
	-	Cross-sectional study on 530 MS users, 528 males and 2 females (age not reported); took MS tea 1–10 times daily for 4–5 years	Increased alertness, dizziness, sedation, hot sensation, hallucination, vomiting, hostility, aggression, excessive tearing, inability to work, aching of muscles and bones, jerky movement of limbs, loss of appetite, weight loss, insomnia, malaise, and restlessness	[47]
	Amphetamine, haloperidol, diphenhydramine, codeine, depressants, unknown chemical substance	Retrospective study on 52 participants with a median age of 30.5 years (range: 2 days–81 years), 84.6% males and 15.4% females	Seizure, nausea, alteration of consciousness, contraction, confusion, headache, dizziness, syncope, myalgia, insomnia, fatigue, loss of appetite, agitation, tremor, ataxia, dystonia, and neonatal withdrawal	[48]
	Wild dagga, wormwood, alprazolam, synthetic cannabinoid, synthetic tryptamine, alcohol, methamphetamine, risperidone	14 kratom exposures reported to the Texas Poison Center Network of 11 males and 3 females aged between 18 and 48 years	Agitation, nausea, vomiting, confusion, tremor, diaphoresis, drowsiness, hallucinations, mydriasis, and abdominal pain	[49]
	Alcohol	Qualitative study on 34 male participants: 22 regular users chewed 10–80 leaves/day for 3–50 years; 6 occasional users chewed 1–20 leaves/day for 1–6 years; 3 non-users; and 3 ex-users	Loss of appetite, craving to eat kratom, withdrawal, compulsion, impaired control, preoccupation, loss of weight, muscle/bone/back/joint aches, cramps/numbness, anxiety, depressed mood, dysphoria, moodiness, annoyance, restlessness, irritability, autonomic nervous system hyper/hypoactivity, chills, sneeze, cough, illness/catch cold, sleepy, yawning, watering eyes, runny nose, sticky mouth, disturbances of behaviors and cognitive functions, and fatigue	[50]
	Mitragynine (291.9 mg/day)	Cross-sectional survey on 293 male kratom users with a mean age of 28.9 years, 153 medium-term users and 140 long-term users; up to ≥3 × 350 mL/day of kratom fresh juice	Sleeping difficulty, decreased appetite, nausea, vomiting, muscle spasm, sweating, fever, abdominal pain, headaches, hot flashes, hiccups, shakiness or tremors, severe muscle pain and cramps, nervousness, sadness, restlessness, anger, tension, depressed mood, and craving	[51]
	-	Qualitative study on 168 kratom users: 109 males, 13 females, and 44 others (sex not specified)	Warmth/tingling, nausea/stomachache, alternating chills/sweats, dizziness/unsteadiness, vomiting, itching, numbness in mouth/throat, visual alterations, and sedation	[52]
	Alcohol, other botanicals, benzodiazepines, narcotics, acetaminophen	660 calls about kratom exposure received by U.S. poison centers, the median age was 28 years (range = 2 months–69 years, data available for 604 subjects), 472 males and 186 females (data available for 658 subjects)	Agitation, irritability, drowsiness, and nausea	[53]
	-	Cross-sectional study on 526 kratom users with a mean age of 51.8 years; took 3–17 leaves/day	Craving-fatigue syndrome, mood symptoms, insomnia, and physical sickness	[54]
	Tramadol, codeine, morphine	Case reports of two kratom-addicted subjects, a 60-year-old female who took 0.25 ounces of kratom every 4 h for several months, and a 57-year-old male	Dependence, tolerance, irritability, increased pain, anxiety, edginess, and leg shaking	[55]
	-	Retrospective study on 150 male participants with a mean (±SD) age of 34.4 (±11.2) years; took up to ≥4 × 350 mL/day of kratom fresh juice	Withdrawal symptoms, anxiety, and depression	[56]
	Morphine	Case report of a maternal (a 29-year-old female) and neonatal kratom exposure; took 3 × 18-20 g/day of kratom powder	Anxiety and maternal and neonatal withdrawal	[57]
	-	Case report of a female infant’s NAS, her mother took 3–4 kratom teas/day during her pregnancy	Excessive sucking, irritability, sleeplessness, and withdrawal	[58]
	-	Case report of a kratom overdose and management of withdrawal of a 24-year-old man with Asperger syndrome, depression, and long-term substance dependence; took 600 mg/day of kratom	Unresponsiveness, seizures, and withdrawal	[59]
	Lisdexamfetamine	Case report of a kratom intoxication of a 19-year-old male with ADHD; took several pills (2–8 g) per day for several months	Seizures	[60]
	Benzodiazepines	Case report of a 52-year-old kratom-addicted female with a long-standing history of MDD; took 1 tablespoon 4–6 times per day for 9 months	Increased depression, anxiety, suicidal thoughts, exacerbation of chronic pain issues, dysphoria, nausea, muscle aches, sweating, goose bumps, and insomnia	[61]
	-	Cross-sectional study on 95 male participants: 70 kratom users with a mean (±SD) age of 28.8 (±5.3) who took 3 glasses of kratom tea/juice daily, and 25 healthy controls with a mean (±SD) age of 25 (±7.6)	Visual memory and new learning impairment	[62]
	-	Cross-sectional study on 150 male kratom users with a mean (±SD) age of 34.4 (±11.2) years; took up to ≥3 glasses/day for more than 6 years	Auditory, visual, tactile, and olfactory hallucinations, persecutory, reference, control and grandiose delusion thought broadcasting, and withdrawal	[63]
	Alcohol, clonazepam, cocaine	Brief report on 2321 kratom exposures, of which 4 cases of NAS and 2 deaths were reported, and 4 deaths for which kratom was listed as a cause or contributing factor to the death	Agitation, drowsiness, confusion, seizure, withdrawal, hallucinations, respiratory depression, coma, and cardiac or respiratory arrest	[64]
	Alcohol, other unknown substances	Case reports of kratom intoxications with 4 males with a mean age of 43.5 years (range = 44–67); case 2 took 4 × 10 g/day of kratom over 24 h	Seizure, sedation, withdrawal symptoms, and dependence syndrome	[65]
	Opiate, methamphetamine, methadone	Cross-sectional survey on 163 regular kratom male users with a mean (±SD) age of 37.1 (±10.9) years; took 1–3 glasses/day of kratom tea for more than 6 years	Weight loss, physical pain, loss of appetite, fatigue, craving for opioid, insomnia, and depression	[66]
	-	Case reports of two kratom intoxications: a 27-year-old male with bipolar disorder and schizophrenia who took 5 g/day of kratom for 3 years, and a 26-year-old female with no history of mental health disorders who took 2–3 pills/day (20–30 g of kratom/day) for 2 years	Restlessness, generalized body aches, overwhelming anxiety, thoughts of suicide, hallucinations, and withdrawal symptoms	[67]
	Dextroamphetamine	Case report of a 22-year-old male with ADHD and kratom use disorder; took kratom tea every 2 h daily (30 g at maximum dosage) for 2 years	Tolerance, irritability, and withdrawal symptoms	[68]
	Diphenhydramine	Case report of a 27-year-old male with a history of ANX, ADHD, benzodiazepine, and opioid use disorders; took up to 4 × 8 mL/day bottles of kratom for 1.5 years	Tonic-clonic seizures	[69]
	Alcohol, antidepressants, benzodiazepines, cannabis, cocaine, hallucinogens, opioids (prescribed or illicit), tobacco	Online anonymous cross-sectional survey on 2798 kratom users with a mean (±SD) age of 40.2 (±11.8) years, 1099 males and 1699 females; took up to 3 × 1–6 g/day of kratom	Sickness, dizziness, alertness, anxiety, sleepiness, and withdrawal symptoms	[70]
	-	Cross-sectional quantitative study on 356 participants, 137 kratom users and 219 non-users aged between 18 and ≥37 years, 212 males and 144 females	Addiction, withdrawal symptoms, and impaired social functioning	[71]
Mitragynine	Scopolamine	Animal study on 36 male mice from the ICR strain: 18 mitragynine-treated mice administered with 5, 10, or 15 mg/kg IP of for 28 days; 6 scopolamine-treated mice; and 12 HC	Impaired working memory function and reduction in locomotory activity	[72]
	7-hydroxymitragynine (0.15 mg/L in blood, 2.20 mg/L in urine), zopiclone, citalopram, lamotrigine	Fatal mitragynine intoxication of a middle-aged male, found with 1.06 and 3.47 mg/L of mitragynine in blood and urine, respectively	CNS depression	[73]
	Morphine, methamphetamine,	Animal study on male C57BL/6 mice; administered with 1, 5, 10, or 20 mg/kg IP 30 min before or immediately after locomotor and consolidation tests, 30 mg/kg IP for 14 days for the withdrawal induction and for 28 days for the chronic study	Decreased activity in the δ, θ, and β, but not α band in the hippocampus and withdrawal	[74]
	Morphine, penicillin G procaine, propofol, xylazine, ketamine, naltrindole, naloxonazine	Animal study on 39 male Fischer 334 rats; administered with 25, 50, 100, or 150 µg of mitragynine and 2.5, 5, 10, or 20 μg of 7-hydroxymitragynine	Increased morphine self-administration after 7-hydroxymitragynine treatment	[75]
	Morphine, urethane, xylocaine	Animal study on 60 male Sprague Dawley rats and other set of animals used for in vivo electrophysiological studies; administered with 1.0, 5.0 and 10.0 mg/kg of mitragynine IP	Impaired acquisition of spatial learning and significant synaptic depression in the hippocampal region at high mitragynine doses	[76]
*Panax ginseng* C. A. Mey	Fluvoxamine, venlafaxine	Case report of a 47-year-old female with chronic depression; took 200 mg of ginseng extract within 24 h	Hypomania	[77]
	-	Case report of a 26-year-old male with history of suicidal thoughts; took 250 mg/day of Chinese ginseng for two weeks	Restless, insomnia, agitation, disorganization	[78]
	Contraceptive pill, ginsenosides (4%)	Placebo-controlled, clinical study on 20 participants with (mean (±SD) age of 20.6 (±4.2), 10 males and 10 females; administered with placebo and 5 × 200, 400, or 600 mg of ginseng extract with a 7-day wash-out between administrations	Decreased speed attention	[79]
	*Ginkgo biloba* L., contraceptive pill.	Placebo-controlled, clinical study on 20 participants with a mean (±SD) age of 20.6 (±4.2), 10 males and 10 females; administered with placebo and 5 × 200, 400, or 600 mg of ginseng extract with a 7-day wash-out between administrations	Decreased speed attention	[80]
	Clomipramine	Case report of ginseng intoxication of a MDD 56-year-old woman; took 300 mg/day of root extract for 2 weeks	Hyperactivity, insomnia, dysphoria, and verbal and physical aggressiveness	[81]
	*Ginkgo biloba* L., vitamins and minerals, cold-liver oil, primrose oil, caffeine, alcohol	Randomized, double-blind, placebo-controlled clinical study on 70 post-menopausal females, 12 ginseng-receiving subjects with a mean (±SD) age of 58.4 (±1.0) years and 14 placebo-receiving subjects with a mean (±SD) age of 57.4 (±0.7) years; administered with 2 × 100 mg/day of ginseng extract for 12 weeks	Slight depression (one case)	[82]
	Ginsenosides (27–30%)	Single-blind, placebo-controlled clinical study on 14- and a 17-year-old ADHD males; administered with 2 × 250 mg/day of ginseng extract	Mild sedation	[83]
	Rg1, Rb1, Rg3, Re, Rc, Rb2, Rd, Rf, Rh1, Rg2s, other minor ginsenosides, escitalopram, venlafaxine, paroxetine, duloxetine, citalopram, bupropion, fluvoxamine, imipramine, trazodone	Open-label study on 35 MDD females with a mean (±SD) age of 45.1 (±9.5) years; administered with 2 g at day 1 of red ginseng extract titrated at 3 g/day for 4 weeks and maintained for another 4 weeks	Headache, insomnia, and hypersomnia	[84]
	Cannabis	Case reports on two ginseng intoxications of 23- and a 79-years-old; took ginseng ≅ 15 and ≅ 20 g/day, respectively	Racing thoughts, anxiety, irritable mood, labile affect, disorganized thought process, constant distraction, and auditory hallucinations,	[85]
	Chemotherapies, ginsenosides, malonylginsenosides	Open-label study on 30 patients with cancer-related fatigue and under chemotherapy with a median age of 58 (range = 48–68) years, 15 males and 15 females; administered with 2 × 400 mg/day ginseng root extract for 4 weeks	Pain, nausea, cognitive disturbance, and seizure	[86]
	Multivitamins, herbal supplements	Case report of a 42-year-old female with no history of psychiatric issue or illicit drug use	Psychosis and confusion	[87]
	-	Case report of a ginseng intoxication (age, sex, and dose not specified)	Mania	[88]
	Methadone	Randomized, double-blind, placebo-controlled clinical study on 74 opioid-addicted participants under methadone maintenance treatment, 48 males with a mean age of 40.64 years and 26 females with a mean age of 39.0 years; administered with 4 × 250 mg of root powder	Sleepiness and agitation	[89]
	-	Case report of a 47-year-old male with no history of psychiatric disorder; took 600 mg/day ginseng to increase energy and sexual activity	Nervousness, aggressive behaviors, irritability, increased psychomotor activity, decreased sleep, and excessive talkativeness	[90]
	Eicosapentaenoic acid, docosahexaenoic acid, Rg1, Rb1, Rg3	Open-label study on 40 ADHD children with a mean (±SD) age of 8 (±1.45) years 31 males and 9 females; administered with 3 mg/day ginseng extract for 12 weeks	Transitory headache (one case)	[91]
*Panax quinquefolius* L.	Phenelzine, bee pollen, other natural medications	Case report of a 42-year-old female with chronic depression, but no history of manic episodes	Mania and hallucinations	[92]
	*Ginkgo biloba* L., ritalin, efamol	Open-label study on 36 ADHD children with a mean (±SD) age of 10.2 (±3.7); administered with 2 × 200 mg/day of ginseng extract for 4 weeks	Increased impulsiveness, hyperactivity and aggressiveness, headache, and tiredness	[93]
	Tamoxifen, other estrogenic botanical supplements	Multicenter, prospective study on 788 with breast cancer; 103 used ginseng after cancer diagnosis	Fatigue	[94]
*Pausinystalia johimbe* (K. Schum.) Pierre ex Beille (yohimbine)	Corynanthine (5, 20 mg/kg)	Animal study on 101 male Sprague Dawley rats; administered with 5 or 20 mg/kg of yohimbine IP	Decrease in spontaneous locomotor activity, overreaction to external stimuli, violent tremors, convulsions, abnormal posture, and decrease in body temperature	[95]
	Xylazine, Doxapram, 4-aminopyridine	Animal study on 82 red deer (*Cervus elaphus* L.), administered with 0.063, 0.124, 0.125, 0.128, 0.189, 0.20, 0.21, 0.23, 0.24, 0.25, or 1.00 mg/kg of yohimbine IV	Rapid reaction to external stimuli, anxiety, and nervousness	[96]
	Desipramine, bupropion, trazodone	Three case reports of manic symptoms following yohimbine administration of three psychiatric patients: a 41-year-old male who took 10 mg of yohimbine orally, and 20- and 43-year-old females who took 20 mg of yohimbine in two sessions and one session, respectively	Tremors, nausea, generalized restlessness, diaphoresis, and palpitations	[97]
	-	Randomized, double-blind, placebo-controlled study on 59 participants: 39 drug-free patients with agoraphobia and panic attacks—11 males with a mean (±SD) age of 36.8 (±8.2) years and 29 females with a mean (±SD) age of 38.8 (±10.1) years; and 20 HC—9 males with a mean (±SD) age of 33.3 (±7.3) years and 11 females with a mean (±SD) age of 43.3 (±7.4) years; administered with 4 × 5 mg of yohimbine capsules	Anxiety, nervousness, hot and cold flashes, restlessness, tremors, and piloerection	[98]
	Acetylsalicylic acid	Case report of an acute yohimbine intoxication of a 16-year-old female; took 250 mg of powdered yohimbine	Weakness, generalized paresthesia, loss of coordination, dissociative state, severe headache, dizziness, tremors, decreased hearing, nausea, diaphoresis, and intermittent palpitations	[99]
	-	Randomized, double-blind, placebo-controlled study on 88 participants: 68 agoraphobic subjects with panic attacks or panic disorder—19 males with a mean (±SD) age of 38 (±9) years and 49 females with a mean (±SD) age of 40 (±1) years; and 20 HC—9 males with a mean (±SD) age of 34 (±8) years and 11 females with a mean (±SD) age of 43 (±7) years; administered with 4x5 mg of yohimbine capsules	Increased rate of panic attacks, anxiety	[100]
	-	Animal study on male hooded Lister rats; administered with 2.5 or 5 mg/kg of yohimbine IP for acute exposure evaluation and 2.5 or 5 mg/kg daily for 5 days for chronic exposure evaluation	Decreased locomotory activity and increased anxiety	[101]
	-	Randomized, double-blind, placebo-controlled study on 65 participants: 45 depressed subjects with a mean (±SD) age of 41 (±13) years—17 males and 28 females; and 20 HC with a mean (±SD) age of 39 (±9) years—9 males and 11 females; administered with 4 × 5 mg of yohimbine capsules	Drowsiness, nervousness, anxiety, fear, sadness, depression, nausea, tremors, hot and cold flashes, and muscle aches	[102]
	Amitriptyline	Animal study on 12 male Sprague Dawley rats; administered with 5 mg/kg IP weekly for 6 weeks	Profound hypothermia	[103]
	-	Animal study on six unrestrained bonnet macaques (*Macaca radiata* É. Geoffroy); administered with 0.10, 0.25, 0.50, or 0.75 mg/kg (0.93–9.74 mg) of yohimbine orally	Distress symptoms, freezing behaviors, clonic jaw movements, excessive yawning, and sexual arousal	[104]
	Clonidine	Randomized double-blind placebo-controlled study on 53 participants: 38 subjects with panic disorders with a mean (±SD) age of 35 (±2) years—11 males and 27 females; and 15 HC with a mean (±SD) age of 30 (±3) years—5 males and 10 females; administered with 0.4 mg/kg of yohimbine over 10 min	Anxiety and panic attacks	[105]
	Glyburide, alcohol	Case report of an acute yohimbine intoxication of a diabetic 63-year-old man; took 100 × 2 mg of yohimbine tablets in about 90–120 min	Anxiety and alertness	[106]
	Tolazoline, xylazine	Animal study on four healthy horses—three mares and one gelding; administered with 0.025 mg/kg of yohimbine every 2 min until a maximal dose of 2.0 mg/kg	Agitation, muscular tremors, mild excitement, and reduction in xylazine-mediated sedation	[107]
	-	Clinical study on eight narcoleptic participants, four males with a mean age of 43.5 years (range 22–64) and four females with a mean age of 26 years (range 21–39); administered with 2.7, 5.4 mg of yohimbine tablets twice daily up to 16.2 mg/day	Insomnia, tremors, and flushing	[108]
	Fluoxetine, TCAs, propranolol, benzodiazepines	Four case reports of a 44-, two 45-, and a 53-year-old males with PTSD; took “high” quantity of over-the-counter yohimbine	Sexual arousal, sweating, shaking, panic attacks, and flashbacks	[109]
	[^15^O] H_2_O	PET scans in a single-blind fixed-order study on nine participants with a mean age of 30.7 years—three males and six females; administered with 0.15 mg/kg up to a maximum dose of 10 mg of yohimbine over 3 min	Panic attack (one subject) and increased anxiety	[110]
	Morphine, U-50,488, SNC80	Animal study on 318 male OF1 mice and male Sprague Dawley rats; administered with 2 and 4 mg/kg of yohimbine IP	Blockage of opioid-mediated spinal antinociception	[111]
	benzodiazepines, [^11^C] flumazenil	Animal study on three adult male rhesus monkeys; administered with 0.4 mg/kg of yohimbine	Increased binding potential for BDZ receptors in the hippocampus and anxiety	[112]
	-	Randomized, double-blind, placebo-controlled study on 17 participants: 8 methadone-maintained subjects with a mean (±SD) age of 38 (±2.6) years—6 males and 2 females; and 9 male HC with a mean (±SD) age of 30 (±3.7) years; administered with 4 mg/kg of yohimbine over 10 min	Precipitation of withdrawal and craving symptoms in methadone patients	[113]
	Xylazine, ketamine, buprenorphine, gentamicin, methamphetamine	Animal study on 73 male Long–Evans rats; administered with 1.25 or 2.5 mg/kg of yohimbine IP after extinction and 0.625 mg/kg after 21–51 days of withdrawal	Reinstatement of extinguished methamphetamine seeking behavior	[114]
	Cocaine, clonidine, RS-79948, flupenthixol	Animal study on 14 adult squirrel monkeys (*Saimiri sciureus* L.); administered with 0.1 and 0.56 mg/kg of yohimbine IP before the reinstatement test	Reinstatement of extinguished cocaine seeking behavior	[115]
	-	Randomized double-blind placebo-controlled study on 13 male participants with a mean (±SD) age of 24.9 (±2.2) years; administered with 0.4 mg/kg of yohimbine IV, procedure repeated after 14 days	Increased restlessness, anxiety, and impaired mood	[116]
	Pentobarbital	Animal study on 12 adult male Sprague Dawley rats; administered with 0.1, 1.0, or 10.0 mg/kg of yohimbine IP	Suppression of P13 and N40 auditory circuitry components at high doses and increment of amplitude at low/moderate doses	[117]
	-	Case report of an acute yohimbine intoxication of a 37-year-old male body builder; took 5 g of yohimbine, found with 5240, 2250, 1530, and 865 ng/mL in blood 3, 6, 14, and 22 h, respectively, after ingestion and 50 mg/L in urine at admission	General malaise, vomiting, loss of consciousness, and tonic-clonic seizures	[118]
	-	Randomized double-blind placebo-controlled study on 24 claustrophobic participants, 12 yohimbine-receiving subjects and 12 placebo-receiving patients, 79% females with a mean (±SD) age of 24.46 (±8.65) years (males not reported); administered with 2 × 5.4 mg of yohimbine pills, procedure repeated after 14 days	Significant improvement in peak fear at the one-week follow-up behavioral assessment	[119]
	Xylazine, ketamine, pentobarbital, chloral hydrate, cefazoline, heparin, heroin	Animal study on 22 male Sprague Dawley rats; administered with 1.25 and 2.5 mg/kg of yohimbine IP 30 min before reinstatement sessions	Stress and reinstatement of extinguished heroin seeking behavior	[120]
	-	Randomized, double-blind, placebo-controlled study on 24 male participants: 12 athletes with an average age of 29 years and 12 untrained HC with an average age of 29.5 years; administered with 0.4 mg/kg of yohimbine IV	Anxiety	[121]
	Nicotine, acamprosate	Randomized, double-blind, placebo-controlled study on 35 participants: 12 acamprosate-receiving subjects with a mean (±SD) age of 44.4 (±1.6) years—11 males and 1 female; and 13 placebo-receiving patients with a mean (±SD) age of 44.1 (±1.9) years—11 males and 2 females; administered with 0.4 mg/kg of yohimbine IV	Increase in alcohol craving symptoms	[122]
	Clonidine, 2-[^18^F]-fluoro-2-deoxy-D-glucose	Randomized, double-blind, placebo-controlled study on 22 participants, 11 patients with IBS with a mean (±SD) age of 40.5 (±12.9) years and 11 HC with a mean (±SD) age of 37.3 (±10.6) years; administered with 40 mg of yohimbine tablet	Increased anxiety and reduced brain activity in the anterior cingulate cortex, amygdala, dorsal brainstem, and posterior insula	[123]
	Nicotine, ketamine, xylazine, buprenorphine, bupivacaine, penicillin	Animal study on 84 Long–Evans juvenile rats; administered with 0.3 and 0.6 mg/kg of yohimbine IP before operative sessions	Increased nicotine self-administration	[124]
	Alcohol, d-Phe CRF	Animal study on 60 rats (strain not reported); administered with 1.25 mg/kg of yohimbine IP before alcohol self-administration and reinstatement sessions	Increased alcohol self-administration and reinstatement of alcohol seeking behavior	[125]
	Caffeine, diphenhydramine, alcohol	Two fatal case reports of acute yohimbine intoxication of 23- and 37-year-old males; found 7400 ng/mL in iliac blood and 5400 ng/mL in heart blood, respectively	Seizures, elevated vitals, and death	[126]
	Pioglitazone, naltrexone, alcohol	Animal study on 193 genetically selected msP male rats; administered with 1.25 mg/kg of yohimbine IP 1 h after naltrexone and before the reinstatement session	Reinstatement of alcohol seeking behavior	[127]
	Nicotine, caffeine,	Randomized, double-blind, placebo-controlled study on 119 participants: 62 cocaine-dependent subjects with a mean (±SD) age of 41.1 (±10.0) years—32 males and 30 females; and 57 HC with a mean (±SD) age of 33.1 (±12.6) years—32 males and 30 females; administered with 21.6 mg of yohimbine capsule	Increase in anxiety and craving in cocaine-dependent subjects	[128]
	-	Animal study on 768 AB strain wild zebrafish larvae, 640 yohimbine-receiving animals; administered with 10, 25, 50, 100, or 200 mg/L of yohimbine solution, and 128 HC	anxiogenic at high concentrations	[129]
	MDMA, SCH-23390, atropine, ketamine, xylazine	Animal study on 67 male Sprague Dawley rats; administered with 2.0 mg/kg before reinstatement test and 5.0 mg/kg daily for 10 days for chronic treatment	Anxiogenic effects and reinstatement of extinguished MDMA seeking behavior	[130]
	naltrexone	Double-blind, placebo-controlled study on 16 healthy women aged between 18 and 32 years: 5 yohimbine + naltrexone-receiving patients, and 11-placebo-receiving patients; administered with 16 mg of yohimbine orally alone and co-administered with 50 mg of naltrexone orally	Agitation, restlessness, anxiety, headaches, nausea, light-headedness, vomiting, weakness, lethargy, tremors, blockage of analgesia in ipsilateral forehead, and increase in electrically evoked pain in forearm	[131]
	Nicotine, THC	Randomized, double-blind, placebo-controlled study on 95 participants: 39 cocaine-dependent subjects with a mean (±SD) age of 41 (±1.7) years—12 males and 27 females; and 56 HC with a mean (±SD) age of 33 (±1.7) years—32 males and 34 females; administered with 21.6 mg of yohimbine prior to two cocaine exposure sessions	Increased impulsivity in HC and slower hit reaction time in female cocaine-dependent subjects	[132]
	Hydrocortisone	Randomized, double-blind, placebo-controlled study on 103 participants with a mean (±SD) age of 24.79 (±0.36) years—51 males and 52 females randomly assigned to four experimental groups (placebo, yohimbine, hydrocortisone, yohimbine + hydrocortisone); administered with 20 mg of yohimbine orally	Decreased memory generalization in females (both yohimbine and yohimbine + hydrocortisone groups)	[133]
	Naltrexone	Randomized, double-blind, placebo-controlled study on 39 participants: 13 yohimbine-receiving patients and 10 placebo-receiving patients—13 males and 10 females aged between 18 and 52 years; 5 yohimbine + naltrexone-receiving females and 11 placebo-receiving females aged between 18 and 32 years; administered with 16 mg of yohimbine alone and co-administered with 50 mg of naltrexone orally	Nausea, headache, malaise, pain, anxiety, and sexual arousal	[134]
	-	Randomized, double-blind, placebo-controlled study on 42 health participants with a mean (±SD) age of 21.29 (±3.27): 21 yohimbine-receiving subjects and 21 placebo-receiving subjects—19 males and 23 females; administered with 20 mg of yohimbine orally 45 min before behavioral test	Anxiety, nervousness, nausea, decreased motor and temporal impulsivity, and increased reflection impulsivity	[135]
*Piper methysticum* G. Forst.	Alcohol, kavain (450.1 mg/L), dihydrokavain (456 mg/L)	Alternated, double-blind, placebo-controlled study on 24 participants with a mean age of 26.7 years (range = 18–53), 10 males and 13 females; 12 subjects received 500 mL of kava juice and 12 received placebo	Body sway and decreased reaction time tasks	[136]
	Valerian	Cross-over study on 24 participants with stress-induced insomnia, 9 males with a mean age of 43.7 years (range 23–65) and 15 females with a mean age of 44.6 (range 30–65) years; administered with 120 mg/day of standardized kava extract for 6 weeks	Dizziness, vivid dreams, and dry mouth	[137]
	-	Randomized, double-blind, placebo-controlled study on 38 participants with a previous history of GAD and a mean (±SD) age of 51.7 (±11.6) years—7 males and 31 females; administered 140 mg/day of standardized kava extract for 1 week and 280 mg/day for the next 3 weeks	Bad taste in mouth, difficulty achieving orgasm, drowsiness, dry mouth, increased appetite, muscle twitching, nausea, spasms or drawing of muscles, sweating, tingling or numbness, and trembling	[138]
	Benzodiazepines, fluoxetine, sertraline	Case report of a kava intoxication of a 45-year-old female with a family history of essential tremor; took 65 mg/day of kava extract for 10 days	Slow saccades, hypophonic speech, generalized postural and tremor at rest, severe generalized rigidity in axial and appendicular muscles, severe akinesia, gait disturbance with lack of balance, and inability to walk	[139]
	-	Randomized, double-blind, placebo-controlled study on 141 participants with neurotic anxiety: 71 subjects with a mean age of 48.8 (range 18–69) years, administered with 3 × 50 mg/day of standardized kava extract for 4 weeks; and 70 placebo-receiving subjects with a mean age of 48.2 (range 18–69) years—36 males and 105 females	Withdrawal, aggravation of anxiogenic symptoms, and tiredness	[140]
	-	Saccade and cognitive study on 28 participants: 11 kava-intoxicated subjects with a mean (±SD) age of 38.1 (±10.3) years and 17 kava-users with a mean (±SD) age of 33.1 (±7.0) years; took 75–375 g of kava powder	Ataxia, tremors, sedation, disorientation, and blepharospasm	[141]
	Kavain (55%)	Randomized, prospective, open study on 68 perimenopausal females: 15 kava-receiving subjects with a mean (±SD) age of 51.5 (±1.1) years were administered with 100 mg/day of calcium plus kava for 3 months; 19 kava-receiving subjects with a mean (±SD) age of 51.1 (±0.8) years were administered with 200 mg/day of calcium plus kava for 3 months; and 34 HC with a mean (±SD) age of 50.2 (±0.6) years	Nausea	[142]
	Flunitrazepam, flumazenil	Animal study on 40 male sleep-disturbed Wistar rats; administered with 10, 30, or 300 mg/kg of a 96% ethanol kava extract	Hypnosis (shorter sleep latency, increase in the total non-REM time)	[143]
	-	Case report of an acute kava intoxication of a 37-year-old male; took a “too strong” kava tea	Leg weakness, severe vertigo, nausea, vomiting, diaphoresis, and dizziness	[144]
	*Hypericum perforatum* L., alcohol, caffeine	Randomized, double-blind, placebo-controlled study on 24 participants with MDD and a mean (±SD) age of 42.9 (±8.8) years—20 males and 38 females; took 3 × 2.66 g/day of herbal kava tablets for 10 months	One case of withdrawal	[145]
	Dihydrokavain (26%), kavain (21%), dihydromethysticin (18%), methysticin (14%), yangonin (13%), Desmethoxyyangonin (8%)	Randomized, double-blind, placebo-controlled study on 28 participants with GAD and a mean (±SD) age of 30.1 (±12.4) years—7 males and 21 females; administered with 120 and 240 mg/day of kavalactones in 3 g tablets of standardized extract of kava roots	Increased females’ sex drive and difficulty to reach the orgasm in males	[146]
	Dihydrokavain (26%), kavain (21%), dihydromethysticin (18%), methysticin (14%), yangonin (13%), desmethoxyyangonin (8%)	Randomized, double-blind, placebo-controlled study on 58 participants with GAD and a mean (±SD) age of 30.1 (±8.8) years, 20 males and 38 females; administered with 120 or 240 mg/day of kavalactones in 3 g tablets of standardized extract of kava roots	Significant reduction in anxiety and headaches	[147]
	Anxiolytics, antidepressants, antinausea, antipsychotics, painkillers, stimulants, sedatives, meth/amphetamine, cannabis, cocaine, ecstasy, hallucinogens, opiates, benzylpiperazine, synthetic cannabis	Survey study on 434 participants with a mean (±SD) age of 34.54 (±14.62) years—36.41% males and 63.59% females, 26 kava-users	Driving-impairment	[148]
	Kavalactones	Animal study on adult wild-type short-fin outbred zebrafish with a 1:1 male-to-female ratio; administered with 10, 20, or 50 mg/L of water extract from powdered kava roots for 20 min and 7.5 mg/L for 1 week	Sedation and immobility	[149]
Methysticin	DMSO	Animal study on 18 AD model mice—12 APP/Psen1 strain and 6 wild-type mice; administered with 6 mg/kg of methysticin once a week for 52 weeks	Reduced movements	[150]
*Ptychopetalum olacoides* Benth.	Diazepam, pentylenetetrazol	Animal study on CF1 strain male adult mice parted in eight groups (15–30 animals); administered with 30, 100, or 300 mg/kg of ethanolic extract for 30 min	Reduced locomotion	[151]
	MK801, DMSO, scopolamine, physostigmine	Animal study on 66 CF1 strain male adult albino mice; administered with 50 or 100 mg/kg of standardized ethanolic and 100–800 mg/kg once a day for 21 weeks	Impairment of both short- and long-term memories	[152]
*Sceletium tortuosum* (L.) N. E. Brown	-	Randomized, double-blind, placebo-controlled study on 21 participants with a mean (±SD) age of 54.6 (±6.0), 9 males and 12 females; administered with 25 mg/day of standardized extract (Zembrin^®^) for 3 weeks	Headache, blurred vision, poor hearing, nausea, vomiting, appetite increase, muscles rigidity, drowsiness, confusion, concentration difficulty, memory problems, depression, anxiety, and ataxia	[153]

AD—Alzheimer’s disease; ADHD—attention deficit and hyperactivity disorder; ADD—attention deficit disorder; BQ—betel quid; CA—Citrus aurantium; CNS—central nervous system; CPZ—cuprizone; EO—essential oil; HC—healthy controls; IBS—irritable bowel syndrome; IP—intraperitoneally; IV—intravenously; MDD—major depressive disorder; MS—Mitragyna speciosa; MSA—multiple system atrophy; NAS—neonatal abstinence syndrome; NPDS—United States National Poison Data System; PAF—pure autonomic failure; PTSD—post-traumatic stress disorder; REM—rapid eye movement; SSRIs—selective serotonin reuptake inhibitors; SNRIs—serotonin and noradrenaline reuptake inhibitors; ST—Sceletium tortuosum; SUD—substances use disorder; TCA—tricyclic antidepressant; TeCA—tetracyclic antidepressant.
